# Reduced risk-seeking in chimpanzees in a zero-outcome game

**DOI:** 10.1098/rstb.2019.0673

**Published:** 2021-01-11

**Authors:** Stefanie Keupp, Sebastian Grueneisen, Elliot A. Ludvig, Felix Warneken, Alicia P. Melis

**Affiliations:** 1 Department of Psychology, University of Warwick, University Road, Coventry CV4 7AL, UK; 2 Experimental Psychology, University College London, 26 Bedford Way, London WC1H 0AP, UK; 3 Department of Psychology, University of Michigan, 530 Church Street, Ann Arbor, MI 48109-1043, USA; 4 Center for Adaptive Rationality, Max Planck Institute for Human Development, Lentzeallee 94, 14195 Berlin, Germany

**Keywords:** chimpanzees, risk, individual differences, zero outcome

## Abstract

A key component of economic decisions is the integration of information about reward outcomes and probabilities in selecting between competing options. In many species, risky choice is influenced by the magnitude of available outcomes, probability of success and the possibility of extreme outcomes. Chimpanzees are generally regarded to be risk-seeking. In this study, we examined two aspects of chimpanzees' risk preferences: first, whether setting the value of the non-preferred outcome of a risky option to zero changes chimpanzees’ risk preferences, and second, whether individual risk preferences are stable across two different measures. Across two experiments, we found chimpanzees (*Pan troglodytes*, *n* = 23) as a group to be risk-neutral to risk-avoidant with highly stable individual risk preferences. We discuss how the possibility of going empty-handed might reduce chimpanzees' risk-seeking relative to previous studies. This malleability in risk preferences as a function of experimental parameters and individual differences raises interesting questions about whether it is appropriate or helpful to categorize a species as a whole as risk-seeking or risk-avoidant.

This article is part of the theme issue ‘Existence and prevalence of economic behaviours among non-human primates’.

## 1. Introduction

Decisions under risk and uncertainty are a ubiquitous part of both human and animal lives, ranging from complex monetary investment decisions in humans to decisions about where and when to forage or how to pursue mating efforts for most other animals. The risky choice is shaped by a number of contextual factors, including the magnitude of available outcomes (i.e. the size or quantity of rewards [1–3]), outcome delays [4–6], choice framing (i.e. whether outcomes are presented as potential gains or losses), the probability of winning or losing a gamble [7–9], social context (for example, playful versus competitive scenarios [10] or bystander versus alone scenarios [11]) and how information is obtained (i.e. via learning-by-description versus learning-by-experience [3,12]).

Studying decision-making under uncertainty in non-human primates, especially other great apes, may be particularly relevant for understanding the evolutionary roots of human decision-making. Such comparative approaches inform hypotheses about whether human risk preferences are related to uniquely human attributes or can be explained by shared and more general cognitive abilities. For example, this approach can provide insights into decision-making biases that may seem irrational at first sight, but actually convey fitness benefits when considering the ecological and other contextual factors in which decisions take place [13–15].

A number of studies have looked at great apes’ responses to risk and uncertainty [10,16–18]. For example, Haun *et al.* [16] presented all four species of great apes with a setup where subjects could choose between a safe reward option (a small piece of banana, which they saw the experimenter put under the ‘safe cup’) and a risky option, which consisted of a variable number of ‘risky cups’ that could potentially contain a larger piece of banana (the experimenter hid one larger piece under one of 1, 2, 3 or 4 risky cups behind a visual barrier out of sight of the subjects). They found that great apes adaptively adjusted their risk-taking strategy, choosing the safe option more frequently as the reward size of the safe option relative to the risky option increased and as the probability of success of the risky option decreased. In addition, chimpanzees and orangutans were generally risk-seeking, whereas bonobos were more risk averse.

Several other studies presented chimpanzees and bonobos with a two-cup choice paradigm to assess risk preference. In these studies, one cup yielded a consistent safe reward (for example, a medium amount of food or one piece of a medium-preferred type of food), whereas the other cup yielded a worse or better outcome with a 50/50 chance [10,17–19]. These studies consistently found that chimpanzees were risk-seeking overall, choosing the variable reward option, whereas bonobos went for the safe option more often. This divergence is somewhat surprising because the two species are closely related and have similar cognitive abilities [20]. One prominent ultimate-level explanation for such species differences in risk tolerance under similar conditions points to differences in their natural feeding ecologies [13,14,19,21,22]. Reliance on naturally abundant and reliable food sources may allow animals to avoid risky foraging strategies and lead to generally risk-averse choices. By contrast, a more unpredictable and patchy feeding ecology may force animals to engage in risky foraging which in turn might shift their cognitive strategies towards risk-seeking in general. The reported differences in risk preferences between chimpanzees and bonobos fit this pattern, with chimpanzees facing a more unpredictable feeding ecology than bonobos under natural conditions (e.g. [19]).

In humans, risky choice is often modulated by the magnitude of the extreme outcomes—the largest and smallest rewards encountered in a choice situation [7,9,19]. One key finding is that people overweight extreme outcomes in both memory and choice [3]: they choose as if they attribute a higher probability to the values at the high and low edges (i.e. the extremes) of the experienced range of outcomes. For example, study participants judged extreme outcomes to have occurred more often than moderate outcomes (when in fact they had been presented equally often). When making experience-based decisions, participants expressed more risk-seeking for gains than losses, and this pattern was dependent on the relative range of the experienced values—people made more risky choices when presented with a set of high-value gain decisions (e.g. fixed +60 versus risky +40/+80) than when presented with a set of low-value gain decisions (e.g. fixed +20 versus risky 0/+40) (for an overview, see [3]).

Interestingly, pigeons behaved according to predictions of this extreme-outcome rule as well [2]. Recent findings, however, indicate that pigeons might particularly avoid zero outcomes rather than overweight extreme outcomes in general, whereas humans treat zero outcomes like other extreme small outcomes [1]. Pisklak *et al.* [1] propose that pigeons treat a risky option with the possibility of a zero outcome as an option that sometimes yields a delayed reward rather than as an option with the possibility of a zero outcome. In delay discounting tasks, subjective reward value decreases rapidly for pigeons [23–25], which might explain their avoidance of zero-outcome options: they avoid the possibility of having to wait for their reward. By contrast, reward value decreases more slowly for great apes, including humans, and they can wait longer for ‘larger-later’ outcomes [25–27].

To our knowledge, neither the outsized impact of extreme outcomes in relation to the presented range of possible outcomes nor a zero-avoidance effect has been systematically tested in non-human primates. Nearly all of the previous risk-taking studies with chimpanzees used small amounts (e.g. only one piece of food) as the unlucky outcome. We know of only one study with chimpanzees where the variable (i.e. more ‘risky’) option sometimes yielded a zero outcome: Proctor *et al*. [28] devised a non-human primate version of the ‘Iowa Gambling Task’—a standard task in psychology to study decision-making in humans—with additional conditions to disentangle reward maximization and risk avoidance. In one condition where both options yielded the same average payoff, chimpanzees were risk-avoidant on average. Chimpanzees' avoidance of the more variable option may have been related to the possibility of getting nothing in a few cases (risk of zero was 10% for the high-variance stack) or may be the result of differences in experimental procedures compared with previous studies (for example, having two static stacks of pre-baited cups to choose from, as in the Primate Gambling Task, is different from having an experimenter set up and re-bait two cups repeatedly for each choice trial). In any case, this finding provides an interesting first indication that the possibility of getting nothing might elicit different responses, namely playing it safer, compared with small-reward gambles in chimpanzees.

Despite chimpanzees’ ability to delay gratification and control their impulsivity to point at ‘smaller-sooner’ options, outcomes yielding zero might have a special standing for them, considering chimpanzees have evolved in a risky feeding ecology. While risk-seeking might maximize chimpanzees' survival chances in most situations [19], it might be more important to avoid the possibility of a zero outcome than to avoid a potential small, but non-zero, outcome. Although investing in gambles with a potential zero outcome might occur less frequently than opting for risky alternatives that yield at least small rewards, chimpanzees do occasionally engage in activities that bear the risk of receiving nothing. For example, when chimpanzees take part in solo and cooperative hunting activities, a hunt might not be successful or others might not share. Also, in the social domain, investing in social partners may bear the risk of getting nothing if others do not reciprocate. In captive settings, pairs of chimpanzees are willing to cooperate even when the distribution of rewards is unequal, i.e. when one partner gets more than the other [29,30]. Cooperation breaks down, however, if one partner can never profit [31,32]. Campbell *et al.* [33] recently showed that the same applies to groups of three chimpanzees (better paralleling the hunting scenario): they were willing to cooperate when two partners received three grapes and the third partner only received one grape, but cooperation broke down when not every trial was rewarded with at least one piece of food for each partner.

In the current study, we examined two aspects of chimpanzees’ risk preferences: First, we asked whether setting the value of the non-preferred outcome of a risky option to zero changes chimpanzees' risk preferences relative to previous findings. Second, we tested whether individual risk preferences are stable across different methods of elicitation. In the first experiment, we followed the experimental procedure of Heilbronner *et al.* [19], but introduced the possibility of a zero outcome. In our task, chimpanzees chose between two cups—one of which (safe) always covered three pieces of food, while the other one (risky) covered either six pieces or zero pieces of food with a 50/50 chance. This shift allowed us to compare chimpanzees’ risky choice when an unlucky gamble can lead to one (Heilbronner *et al*.) or zero (current study) pieces of food, and thus to assess whether chimpanzees' risk-prone choice strategy holds even when they are presented with a gamble that includes the possibility of a zero outcome. If zero-outcome gambles affect risky choice differently from other gambles, we expect the chimpanzees in the current study to be more risk averse than chimpanzees in previous studies. We complemented this with a second experiment, where we applied a titration procedure to assess the average size of the safe option needed to change chimpanzees’ strategy from risk-seeking to risk aversion and *vice versa*.

## 2. Material and methods

### (a) Subjects

We tested 23 chimpanzees (11 males, 12 females, age range 12–35 years; see also electronic supplementary table S1 for information about the subjects) from Ngamba Island Chimpanzee Sanctuary, Uganda (https://ngambaisland.org/). These are orphaned chimpanzees who were rescued from the illegal bushmeat and pet trade. Throughout the day the entire group (50 individuals) has access to a 95-acre secondary forest on the island to forage and roam freely. The group is additionally fed four times a day with fruits, vegetables, posho (maize flour dish) and porridge; water is available ad libitum. At night the chimpanzees sleep in a large holding facility (542 m^3^) consisting of nine rooms with interconnecting corridors or sliding doors. Testing took place in a familiar room in the holding facility. The chimpanzees were never food-deprived for this study and could stop participating at any time by leaving the testing area and approaching the door to the forest. These chimpanzees frequently participate in cognitive-behavioural testing and are familiar with different experimental setups. They had participated in another risk preference study four months prior to our data collection, which was conducted independently by another team of researchers. In this other study, a different method was used to assess attitudes to risk and uncertainty: reward magnitudes and the presentation procedure of risky choices differed from our study, and no results are available yet. The current research was approved by the University of Warwick research ethics committee and the Chimpanzee Sanctuary and Wildlife Conservation Trust (CSWCT) as well as the Uganda Wildlife Authority and the Uganda National Council for Science and Technology (UWA/COD/96/05). We did not conduct an *a priori* power analysis to determine sample size because we did not know how many subjects we would be able to test in the available time; our goal was to test as many chimpanzees as possible.

### (b) Setup and general procedure

**Figure 1.**
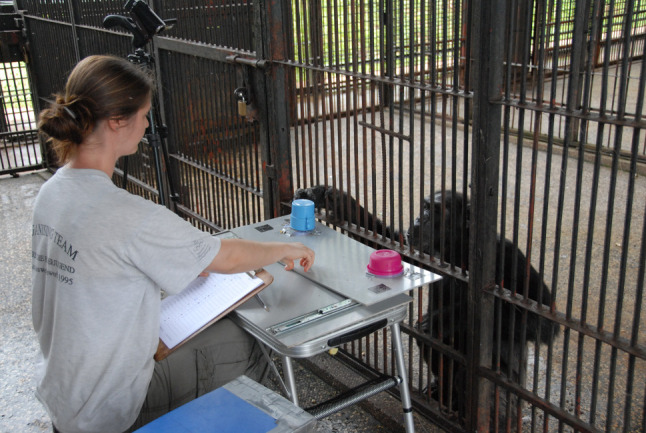
General setup with covered safe option (blue/tall cup) and risky option (pink/shallow cup). The centres of the two plastic lids on which the options were presented were 42 cm apart.

Chimpanzees were tested individually in their familiar sleeping rooms. The experimenter (E) presented two choice options hidden under two different cups on a table in front of the testing room (figure 1). The ‘safe’ option always held three pieces of apple; the ‘risky’ option either held six pieces of apple or none with a 50% chance of the cup being baited or empty. We chose these reward outcomes to equate the expected value of the risky and the safe option, thereby not potentially biasing the chimpanzees towards the option with a higher overall expected value. Apple is not part of the chimpanzees' normal feeding regime on Ngamba Island. They can only get pieces of apple as rewards during experiments, and it is a highly attractive test treat. Our rewards were thus of similar value to the chimpanzees in the current study as were the half-grape rewards for the chimpanzees in Heilbronner *et al*. [19].

We used two cups of different shape and colour as the safe and risky options. The assignment was the same for all chimpanzees: pink/shallow was the ‘risky cup’, and blue/tall was the ‘safe cup’.^1^ E baited the cups behind an occluder always starting on the left side and always manipulating both sides (even when the risky option was empty) such that the chimpanzee could not determine the amount and location of food items from monitoring E's movement or noise.

A trial began when E lifted the occluder. After 4 s from trial onset, E pushed the table forward, and subjects had 15 s to indicate their choice by pointing to the cup (i.e. extending their fingers through the bars in front of the cup without reaching it) or by touching it. E only looked up when pushing the table forward and ignored all pointing signals that occurred before that moment. We used the first pointing signal after the table was fully extended towards the chimpanzee as the choice criterion. E ignored any prior pointing signals. When the chimpanzee had indicated its choice, E uncovered the chosen option while pulling back the table. If the choice yielded a food reward, E handed it to the chimpanzee and always revealed the other option (i.e. what is often called a full-feedback procedure, [34]). If the other option contained food, E visibly removed that food from the table before preparing the next trial. The next trial began immediately. The distance between the edge of the extended table and the bars was varied depending on the individual chimpanzee's finger reach and its tendency to grab the cups or table when within reach.

### (c) Familiarization and test procedure

The procedure consisted of a three-step familiarization phase and a test phase. Each subject received one session per day. For the familiarization and the test sessions in Experiment (Exp.) 1, we followed the procedure as described in [19].

During the familiarization, we first assessed if subjects could reliably distinguish the relevant quantities presented during the test. These *Numerical-discrimination* sessions consisted of 20 trials each. Chimpanzees could see the food rewards for 4 s after the occluder was lifted. Then E covered the rewards with the appropriate cup (i.e. ‘safe cup’ for three pieces and ‘risky cup’ for six or zero pieces) and pushed the table towards the subject. To pass the numerical-discrimination test, chimpanzees had to choose the larger reward in eight of 10 trials per combination (i.e. ‘three versus zero’ and ‘three versus six’ pieces of food). The position of options was pseudo-randomized to occur equally often on both sides of the table with no combination occurring more than three times in a row. Twenty-one chimpanzees passed the numerical discrimination test after the first session. Only two individuals needed a second session.

Second, we presented an *Introductory* session to familiarize the chimpanzees with the two different cups. This session consisted of 20 trials in which chimpanzees had only one option available. We presented the risky option 10 times (five times with six pieces and five times empty) and the safe option 10 times (always with three pieces). We pseudo-randomized order of the presented option and presentation side, with no combination occurring more than twice in a row. No criterion had to be reached, and each chimpanzee received one Introductory session.

Finally, we presented six *Mixed sessions* during which all trial types were presented. A Mixed session consisted of six Numerical-discrimination trials, four Introductory trials and 10 Choice trials where the chimpanzees could choose between the covered risky and safe options (note that Choice trials were identical to the test trials in Exp. 1 and served to familiarize the chimpanzees with this type of trial). The order of trials and presentation side was pseudo-randomized, with no more than three trials of the same type presented in a row. No criterion had to be reached, and each chimpanzee received six Mixed sessions.

During the main test phase, each subject received sixty test trials, presented in three *Test sessions* with twenty choice trials each (Exp. 1). Each chimpanzee then participated in one *Titration session* of twenty trials, during which the safe option changed as a function of the previous choice (Exp. 2). Test and Titration sessions lasted approximately 10–12 min each and included 10 choice trials where the risky cup was baited with six pieces of food and 10 choice trials during which the risky cup was empty. In Exp. 2, we implemented a titration procedure where the size of the safe option was adjusted according to the choice in the previous trial. By using this titration protocol, we aimed at establishing an indifference point for each chimpanzee, i.e. the reward size at which each chimpanzee switches from risk-seeking to risk aversion (and *vice versa*). In the titration trials, when E lifted the occluder, the risky option was always covered, but the safe option was visible at first (to allow the chimpanzee to take the current size of the safe option into account). After 4 s, E also covered the safe option and pushed the table forward for the subject to make its choice. If the chimpanzee chose the safe option, the safe option was *reduced* by one food item in the next trial. If the chimpanzee chose the risky option, the safe option was *increased* by one food item in the next trial (up to a maximum of six pieces). In Trial 1, the safe option consisted of the usual three pieces. The presentation side of the two options was pseudo-randomized, with both options presented 10 times on each side, and the content of the risky cup was also pseudo-randomized with the high (6) and low (0) outcome each occurring exactly five times on each side.

### (d) Coding

All trials were coded live by the experimenter. A second coder, who was blind to the hypothesis of the study, coded cup choice in 20% of the trials from videotape. Inter-rater reliability was perfect (*N* = 380 trials; 100% inter-observer agreement, Cohen's *κ* = 1).

## 3. Results

### (a) Experiment 1

#### (i) Main findings

In Exp. 1, chimpanzees made 60 choices between a safe and a risky option, where they had a 50/50 chance of getting six versus zero rewards. An initial assessment of the data revealed a strong side bias in nine of the 23 tested chimpanzees, i.e. approximately 40% of the chimpanzees tested. We considered individuals to exhibit a side bias in a given test session if they chose the same side in at least 15 out of the 20 trials and categorized an individual as having a strong side bias when this happened in two or more test sessions. Consistently choosing one side resulted in equal choice of the risky and safe option owing to our counterbalancing of the two options. A side bias can be a sign of indifference to the presented options (in this case, indifference regarding the riskiness of the outcome) or an indicator that the chimpanzees had not learned the association between each cup (pink versus blue) and the associated option (risky versus safe). In the first case, the side bias would adequately reflect that an individual is neither particularly risk averse nor risk-seeking. In the second case, however, it could mean that the choice pattern does not reveal an individual's attitude towards risk, because the individual is unaware of the contingency between the cup and the reward outcome, and rather indicates that the individual fell back to a simplistic choice strategy. To account for the latter possibility, we additionally conducted our analyses on the subset of individuals that did not exhibit a side bias. A Shapiro test indicated that normality assumptions were violated for chimpanzees' risky choice so we proceeded using nonparametric Wilcoxon tests to analyse the data.

**Figure 2.**
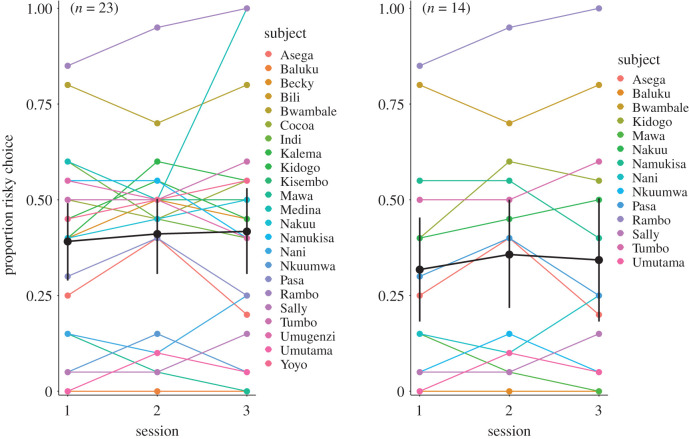
Proportion of risky choices per individual per session in Exp. 1. Left: for the full sample. Right: for the subset with no side bias. Black lines/points represent mean per test session and 95% confidence intervals.

The full sample of chimpanzees (*n* = 23) chose the risky option on 41% of the trials on average and as a group tended to be risk averse as assessed by a Wilcoxon signed-rank test (*z* = −1.79, *p* = 0.076, *r* = −0.37). The 14 unbiased chimpanzees chose the risky option on 34% of the trials (*z* = −1.82, *p* = 0.075, *r* = −0.49). Figure 2 shows individuals’ risky choices across the three test sessions. Notably, there was considerable variation, ranging from one individual who almost always chose riskily across all three sessions to one individual who never chose riskily. We found no general pattern towards more risk-seeking or risk aversion with increasing test experience for the group as a whole (see electronic supplementary material). For results of the effect of age and sex, see electronic supplementary material. No formal analysis was performed on the effect of rank on choice because this assessment is based on our and the animal keepers' knowledge of the chimpanzees rather than systematic observational data.

We were also interested in whether the choice was affected by the outcome of previous risky choices. To this end we assessed if chimpanzees were more likely to choose the risky option when they had won (lost) in the previous risky choice, when this choice happened in the last (one-step-back) or penultimate (two-step-back) trial. The number of observations per individual in these subsets naturally differed because they depended on individual choices. We used generalized linear mixed models to analyse the data [35]. Our full model included the previous outcome (win/lose) as a fixed effect and subject ID as a random effect. To assess the effect of the previous outcome on choice in each trial we compared this with a null model only including the random effect. All models were fitted using the function *glmer* of the package *lme4* [36].

#### (ii) One-step-back analysis

Following a risky choice in the previous trial, chimpanzees (*n* = 23) chose the risky option after a previous win (win–stay) on 24.7 ± 4.5% (here and in the following, 95% CI values are reported) of trials, and, after a previous loss (lose-stay), 20.9 ± 6.2% of the time. They chose the safe option after a previous win (win–shift) on 23.0 ± 5.6% of the trials after a risky choice, and, after a previous loss (lose–shift), 31.5 ± 8.5% of the time (see electronic supplementary material, table S2 for details of individual choice patterns). Overall, the outcome of a risky choice in the previous trial did not predict choice on subsequent trials (*χ^2^* = 0.301, d.f. = 1, *p* = 0.58, conditional *R^2^* = 0.153).

Following a risky choice in the previous trial, the unbiased chimpanzees (*n* = 14) chose the risky option after a previous win (win–stay) on 22.5 ± 7.4% of the trials and, after a previous loss (lose–stay), 17.3 ± 9.8% of the time. They chose the safe option after a previous win (win–shift) on 23.8 ± 9.1% of all trials, and, after a previous loss (lose–shift), 36.5 ± 13.8% of the time. Overall, the outcome of a risky choice in the previous trial did not predict choice on subsequent trials for the unbiased subset either (*χ*^2^ = 0.35, d.f. = 1, *p* = 0.55, conditional *R^2^* = 0.277).

We also conducted a Wilcoxon test for paired samples to see if overall chimpanzees were more likely to choose according to a ‘win–stay/lose–shift’ strategy than a ‘win–shift/lose–stay’ strategy. Chimpanzees (*n* = 23) used a win–stay/lose–shift strategy 56.2 ± 7.1% of the time and a win–shift/lose–stay strategy 43.8 ± 7.1% of the time which was not statistically significant (*z* = 1.41, *p* = 0.16, *r* = 0.29; see also electronic supplementary material, figure S1). The same pattern arose in the unbiased set: chimpanzees (*n* = 14) used a win–stay/lose–shift strategy 58.8 ± 11.2% of the time and a win–shift/lose–stay strategy 41.2 ± 11.2% of the time (*z* = 1.22, *p* = 0.24, *r* = 0.33).

#### (iii) Two-step-back analysis

Following a risky choice in the penultimate trial, chimpanzees (*n* = 23) chose the risky option after a previous win (win–stay) on 21.2 ± 5.8% of the trials, and after a previous loss (lose-stay) 21.9 ± 5.7% of the time. They chose the safe option after a previous win (win–shift) on about 24.5 ± 6.2% of the trials, and, after a previous loss (lose–shift), 32.6 ± 7.8% of the time (see electronic supplementary material, table S3 for details of individual choice patterns). Overall, the outcome of a risky choice in the earlier trial did not predict choice behaviour in the next trials (*χ^2^* = 1.56, d.f. = 1, *p* = 0.21, conditional *R^2^* = 0.170).

Following a risky choice in the penultimate trial, the unbiased chimpanzees (*n* = 14) chose the risky option after a previous win (win–stay) on 17.3 ± 9.3% of the trials, and after a previous loss (lose-stay) 19.5 ± 9.5% of the time. They chose the safe option after a previous win (win–shift) on 26.5 ± 10.1% of the trials after, and, after a previous loss (lose–shift), 36.7 ± 13.1% of the time. Overall, the outcome of a risky choice in the earlier trial did not predict choice behaviour in the next trials (*χ*^2^ = 0.394, d.f. = 1, *p* = 0.53, conditional *R^2^* = 0.318).

Again, we also looked whether the chimpanzees were more likely to choose according to a win–stay/lose–shift than a win–shift/lose–stay strategy but we did not find a significant difference between the two strategies. Chimpanzees (*n* = 23) used a win–stay/lose–shift strategy 53.6 ± 5.3% of the time and a win–shift/lose–stay strategy 46.4 ± 5.3% of the time (*z* = 1.40, *p* = 0.17, *r* = 0.29). In the unbiased set, chimpanzees (*n* = 14) used a win–stay/lose–shift strategy 53.3 ± 8.5% of the time and a win–shift/lose–stay strategy 46.7 ± 8.5% of the time (*z* = 0.87, *p* = 0.41, *r* = 0.23).

Taken together, these results are consistent with previous findings where chimpanzees did not show a win–stay/lose–shift strategy in risky-choice tasks [17,18]. Chimpanzees can, in principle, employ this strategy, as apparent in an experiment where they could repeatedly choose between two conspecific collaborators [37]. However, they do not seem to base their risky choices on such a strategy.

### (b) Experiment 2

In Exp. 2, chimpanzees made 20 choices between a safe option and a risky option. The difference from Exp. 1 was that the size of the safe option was adjusted as a function of the individual's previous choice. This titration procedure provided an additional measure for each individual's risk preference: the size of the safe option that induced an individual switch to a risk-seeking/risk-avoiding choice. The average outcome of the risky option was always three pieces (i.e. six or zero pieces), and the initial safe outcome was three pieces of apple. Thus, an average safe option of more than three pieces indicates a risk-seeking individual, and an average of less than three pieces indicates a risk-averse individual.

Initial assessment of the data revealed a side bias in five individuals, only two of whom had expressed a side bias in Exp. 1. This means that the majority of previously side-biased chimpanzees did not show a side bias in Exp. 2. Importantly, however, the numerical side-bias criterion (for a session of 20 trials this corresponds to choosing one side ≥15 times) might not be ideal given the experimental procedure of Exp. 2. This is because the average amount presented on each side as well as the number of relatively larger/smaller rewards on each side depended on the individual's previous choices and was consequently not as consistently counterbalanced as in Exp. 1. Therefore, we decided to not treat these five individuals as classically side-biased and instead report results for the full sample. For assessment of the relationship between choice behaviour in Exp. 1 and Exp. 2, we additionally report results for the subset of individuals who had no side bias in Exp. 1.

**Figure 3.**
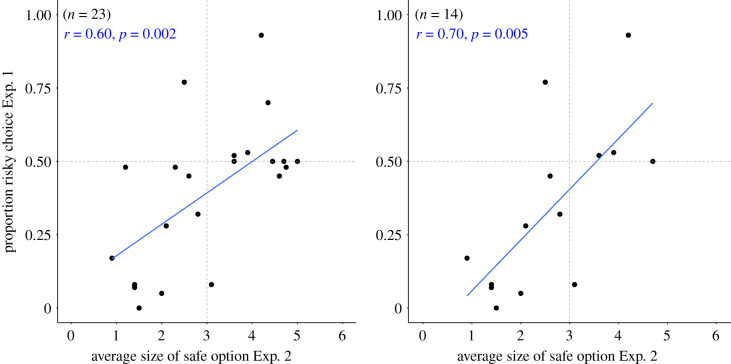
Relationship between the proportion of risky choices in Exp. 1 and the average size of the safe option in Exp. 2. Left: for the full sample. Right: for the subset with no side bias. (Online version in colour.)

The average size of the safe option across 20 trials was 3.1 ± 0.15 pieces for the full sample and 2.6 ± 0.17 pieces for the unbiased Exp. 1 subset. As in Exp. 1, the chimpanzees' risk preferences varied widely across the spectrum, and the group as a whole cannot be easily classified as risk-seeking or risk averse. The range of preferred safe options varied all the way from 0.9 to 5 pieces (see electronic supplementary material, table S4). To capture the degree of stability in individuals’ choices we measured the percentage of choices falling within a 1.5-point range around each individual mean of safe option size. We found that 85% of choices fell within this 1.5-point range (see electronic supplementary material, table S4 for more details). Figure 3 shows how the risky choice in Exp. 1 and the average size of the safe option in Exp. 2 were highly correlated (full sample: *r_τ_* = 0.41, *z* = 2.61, *p* = 0.009; unbiased subset: *r_τ_* = 0.5, *z* = 2.47, *p* = 0.013).

## 4. Discussion

Across two experiments we found less risk-seeking in chimpanzees compared with previous findings [10,16–19]. In Exp. 1, we employed the same basic paradigm that produced risk-seeking in chimpanzees [19] with the crucial difference that the risky option had the possibility of yielding a zero outcome and not just a small reward. Whereas Heilbronner *et al.* [19] found that chimpanzees in their study chose the risky option in 64% of the trials when the unlucky outcome was one piece of food, in the current study chimpanzees chose the risky option in only 41 and 34% (full sample and unbiased sample, respectively) of the trials when the unlucky outcome was zero. Our results also differ from other previous findings, which were obtained with different methodologies, yet presented a picture of chimpanzees as being risk-seeking across a range of different presentation procedures, outcome options and social contexts [10,16–18]. For example, in Haun *et al*. [16], chimpanzees chose the risky option in 100% of the trials when the size of the safe reward was small or medium and still in about 60% of the trials (as inferred from their fig. 2*a*) when the size of the safe reward was large. Rosati & Hare [10] showed that chimpanzees became even more risk prone after manipulating the social context. Specifically, they found that a competitive context (but not a playful one) made chimpanzees more risk prone compared with a neutral control condition, where the chimpanzees had already picked the risky option in more than 60% of the trials. The option of getting nothing, in the current study, might have shifted chimpanzees' preference towards playing it safe.

In Exp. 2, we implemented a titration procedure where the amount of the safe option was adjusted according to the choice on the previous trial to obtain individual indifference points at which each chimpanzee switched between risk-seeking and risk avoidance. The chimpanzees’ behaviour in this titration task indicated mild risk aversion to risk neutrality—again a very different pattern compared with the clearly risk-seeking decisions of chimpanzees in previous studies. The significant correlation between results in Exp. 1 and Exp. 2 (figure 3) indicates that both measures tap into the same underlying decision-making process. Those chimpanzees that were more likely to pick the safe option in Exp. 1 were the same ones that played it safer in Exp. 2 as indicated by their lower average size of the safe option, and, *vice versa*, those that were more likely to pick the risky option in Exp. 1 had a higher average size of the safe option in Exp. 2.

Despite a general group trend towards risk avoidance in Exp. 1, we also found large individual differences in risky choice strategies. These differences were, on first sight, not systematic to obvious characteristics like age, sex or rank, according to our knowledge of these chimpanzees and information from the keepers regarding dominance relationships in the group at the time of data collection. Future research may reveal if risk-taking strategies are related to other individual characteristics such as patience or curiosity, or perhaps to previous experience with experiment participation or other human interactions.

An issue worth considering further is that the procedure in Exp. 1 resulted in a strong side bias for several individuals. As discussed above, this could mean either that these individuals were indifferent to the riskiness of the presented options or that they lacked full comprehension of the consequences of choosing each of the cups. We were surprised by the relatively high number of individuals with a side bias given that Heilbronner *et al*. [19] did not have a similar high proportion of side-biased individuals (four of five chimpanzees preferred the risky option, and all five bonobos preferred the safe option, a result that precludes side bias owing to counterbalanced side presentation of both options). The correlation between Exp. 1 and 2 clearly indicates that the current study consistently captured chimpanzees' risk preferences, which suggests that it was not a lack of comprehension that led to the side bias, but rather risk indifference. Other studies [10,17,18] have used different protocols, which allowed the inclusion of knowledge probes that directly tested for task understanding. For example, Rosati & Hare [17] always baited the safe option in full view of the chimpanzee and then presented a ‘risk outcome’ container, which contained the range of possible risky outcome rewards (for example, a small and a large piece of food). Subjects knew that only one of the items would be hidden under the risky cup, but not which one. After the chimpanzee had inspected the risk outcome container, the risky option was baited hidden from the chimpanzee's view. In order to succeed in comprehension trials, chimpanzees had to switch flexibly between risky and safe options as a function of trial type. For example, if the safe option contained two pieces of food and the risk outcome container contained two pieces of food (‘comprehension-1’ trials, see [17]), the rational choice was to go for the safe option because only one piece of food will be hidden during the baiting of the risky cup. Chimpanzees showed high success rates in those control trials, indicating that they did not struggle to comprehend the paradigm. Side bias was not an issue in our Exp. 2, where the chimpanzees were explicitly shown the current size of the safe option for each trial (as in Rosati & Hare [17]).

The increased risk avoidance of at least some of the chimpanzees in our sample resembles the zero-avoidance strategy of pigeons in previous studies [1]. Pisklak *et al*. [1] raise an interesting point in their discussion of a zero-avoidance effect in pigeons. When confronted with the zero-outcome risky option, pigeons might treat the risky probabilistic reward as a variable delay to reward. Pisklak *et al*. [1] suggested that the risky choice with a zero outcome resembles a self-control/temporal discounting task, where one can get an immediate small reward or a larger reward that sometimes occurs after a delay. Because pigeons show steep discounting functions in intertemporal choice tasks, this fits with the pigeons' avoidance of possible delayed rewards. The same might be true, though perhaps to a lesser extent (as chimpanzees do not show such steep temporal discounting functions, see for example [27]), for the chimpanzees tested in this study. Chimpanzees’ avoidance of the risky option may be related to the delayed larger reward delivery. It will be an interesting question to assess whether individual differences in zero-avoidance (as indicated by a preference for the safe option in the current study) match the individual's behaviour in an intertemporal choice task: those individuals that are more impatient might show more zero-avoidance than those that are better able to delay gratification.

We have considered other explanations why chimpanzees in the current study were less risk-seeking than chimpanzees in the majority of previous studies. One possibility is that our sample might differ systematically from previous samples in important aspects, such as housing conditions, rearing history or test experience. Our sample consisted of sanctuary-housed chimpanzees with an extensive history of cognitive-behavioural testing. Previous studies tested zoo-housed chimpanzees [16,19] but also chimpanzees from another sanctuary population [10,17,18] and all found similarly high levels of risk-seeking. The chimpanzees from these previous studies also had an extensive history of cognitive-behavioural testing. Therefore, unless the chimpanzees from the two sanctuaries are very different, this seems an unlikely explanation. Furthermore, a recent study has shown that chimpanzees from our sample population and chimpanzees from the same zoo population tested by [19] and [16] performed similarly in intuitive statistical reasoning [38]. In sum, we find it unlikely that our results are due to sample differences rather than a zero-outcome effect.

Another possibility is that the lower total gain of our options compared with Heilbronner *et al*. [19] (current study: three versus zero and three versus six; Heilbronner *et al*.: four versus one and four versus seven) might have shifted chimpanzees' preferences toward risk aversion. However, this explanation does not fit well with the finding that various studies, which differed in how and which rewards were presented, consistently reported risk-seeking in chimpanzees. For example, Rosati & Hare [10] found a strong preference for risk-seeking when using different food qualities rather than magnitudes and Haun *et al*. [16] used different sizes of banana slices (one large slice for risky option and one small size for safe option—with the relative size of the safe option varying depending on condition) and also found risk-seeking preferences.

This study contributes towards a fuller picture of understanding chimpanzee risky choice by showing that risk preferences are malleable and are likely influenced by extreme outcomes such as zero; at the same time, we found stability at the individual level when using two different measures. Our finding that a potential zero outcome might have a qualitatively different standing from other small outcomes aligns with findings on chimpanzee behaviour in economic games where they acted like rational maximizers and accepted even the smallest offer as long as it was larger than nothing [39]. The current results indicate that chimpanzees choose more carefully when a situation bears the risk of going empty-handed, whereas they are prone to gamble when they can take home at least one certain piece of food. Gilby & Wrangham [40] found that risk-prone hunting activities were observed more frequently in periods of low nutritional stress. It seems that chimpanzees in the wild do engage in activities of uncertain and potentially zero outcome, but they are more likely to do so when there is ‘insurance’: they do gamble, but only if they cannot go broke; otherwise they play it safe.

Like chimpanzees, humans exhibit individual differences in their risk preferences and many factors influence how risk-seeking we are [41]. One of the key factors that influences how people make risky choices is how the information about the options is obtained [34,42]. For example, humans are typically risk averse for gains and risk-seeking for losses when asked in terms of explicitly described odds and outcomes, but this pattern can reverse when learning from experience [43–45]. Although humans may be used to processing hypothetical and described information in the modern world, we also often fall back to more automatic and evolutionary ancient decision-making processes [46], which might be better captured when learning about risk by experience. Studying decision-making under uncertainty in non-human primates, and in particular, knowing more about the malleability and stability of other great apes' risk preferences, can help us understand the evolutionary roots of this aspect of human decision-making.

Our sample size was large enough to observe considerable variation in individual risk propensities. On a theoretical level, this raises the question if talking about risk preference at the species level is a meaningful concept. In the same way that risk preference cannot be applied as a blanket statement to humans as a species—context, task format and payoff structures matter—the same might be true for chimpanzees and other animals.

## Supplementary Material

Supplementary information

## Supplementary Material

Familiarisation

## Supplementary Material

Individuals

## Supplementary Material

NumericalDiscrimination

## Supplementary Material

RISK_1_AllData

## Supplementary Material

RISK_2_Titration

## Supplementary Material

RISK_3_OneStepBack_binary

## Supplementary Material

RISK_4_TwoStepBack_binary

## Supplementary Material

RISK_Strategies_1stepback

## Supplementary Material

RISK_Strategies_1stepback

## Supplementary Material

CodeBook

## Supplementary Material

Analysis R-code

## Endnote

1 We did not counterbalance cup assignment across chimpanzees because this experiment is part of a test battery to assess individual differences and it was important to provide all individuals with the same stimuli and order of conditions. All individuals, however, participated in Numerical-Discrimination sessions and introductory trials before the test (see test procedure (§2c)). In the numerical-discrimination trials, both cups sometimes contained the bigger (or smaller) amount, so that individuals were rewarded equally often for choosing both types of cups. All subjects passed this phase very quickly, indicating no bias for either cup when they had a clear preference.

## Data accessibility

Supplementary information including data and R-code are included as supplementary material and additionally can be accessed here: https://osf.io/w4bzc/?view_only=0f006aa05c13422ead52019c49d62075.
