# Supplementary material for: Reduced risk-seeking in chimpanzees in a zero-outcome game
Source: Philos Trans R Soc Lond B Biol Sci. 2021 Jan 11;376(1819):20190673. doi: 10.1098/rstb.2019.0673 (PMC7815432; doi:10.1098/rstb.2019.0673)
Supplement: Supplementary information [file rstb20190673supp1.docx]

**Information about individuals**

| Table S1  *Information about subjects (asterisks indicate individuals who had no side-bias in Experiment 1)* | | |
| --- | --- | --- |
| Subject | Sex | Age (years) |
| Asega* | Male | 21 |
| Baluku* | Male | 21 |
| Becky | Female | 28 |
| Bili | Female | 21 |
| Bwambale* | Male | 19 |
| Cocoa | Female | 12 |
| Indi | Male | 20 |
| Kalema | Male | 23 |
| Kidogo* | Female | 35 |
| Kisembo | Male | 20 |
| Mawa* | Male | 23 |
| Medina | Female | 12 |
| Nakuu* | Female | 18 |
| Namukisa* | Female | 21 |
| Nani* | Female | 18 |
| Nkuumwa* | Female | 23 |
| Pasa* | Female | 20 |
| Rambo* | Male | 15 |
| Sally* | Female | 28 |
| Tumbo* | Male | 30 |
| Umugenzi | Male | 22 |
| Umutama* | Male | 23 |
| Yoyo | Female | 21 |

**Choices in earlier trials**

| Table S2  *Overview of choices after previous trial was a risky choice (asterisks indicate individuals who had no side-bias in Experiment 1)* | | | | | | | |
| --- | --- | --- | --- | --- | --- | --- | --- |
| Subject | Total previous risky choices | Previous won | Previous lost | Win-stay | Win-shift | Lose-stay | Lose-shift |
| Asega* | 17 | 12 | 5 | 3 (18%) | 9 (53%) | 1 (6%) | 4 (24%) |
| Baluku* | 0 | 0 | 0 | 0 | 0 | 0 | 0 |
| Becky | 25 | 13 | 12 | 6 (24%) | 7 (28%) | 4 (16%) | 8 (32%) |
| Bili | 29 | 14 | 15 | 9 (31%) | 5 (17%) | 6 (16%) | 9 (32%) |
| Bwambale* | 44 | 23 | 21 | 15 (34%) | 8 (18%) | 18 (41%) | 3 (7%) |
| Cocoa | 29 | 15 | 14 | 10 (34%) | 5 (17%) | 5 (17%) | 9 (31%) |
| Indi | 28 | 14 | 14 | 7 (25%) | 7 (25%) | 6 (21%) | 8 (29%) |
| Kalema | 27 | 13 | 14 | 6 (22%) | 7 (26%) | 10 (37%) | 4 (15%) |
| Kidogo* | 30 | 15 | 15 | 5 (17%) | 10 (33%) | 7 (23%) | 8 (27%) |
| Kisembo | 28 | 14 | 14 | 6 (21%) | 8 (29%) | 8 (29%) | 6 (21%) |
| Mawa* | 4 | 1 | 3 | 1 (25%) | 0 | 0 | 3 (75%) |
| Medina | 40 | 19 | 21 | 14 (35%) | 5 (13%) | 16 (40%) | 5 (13%) |
| Nakuu* | 26 | 11 | 15 | 5 (19%) | 6 (23%) | 9 (35%) | 6 (23%) |
| Namukisa* | 29 | 13 | 16 | 8 (28%) | 5 (17%) | 8 (28%) | 8 (28%) |
| Nani* | 10 | 5 | 5 | 1 (10%) | 4 (40%) | 1 (10%) | 4 (40%) |
| Nkuumwa* | 5 | 2 | 3 | 1 (20%) | 1 (20%) | 0 | 3 (60%) |
| Pasa* | 19 | 9 | 10 | 2 (11%) | 7 (37%) | 3 (16%) | 7 (37%) |
| Rambo* | 53 | 26 | 27 | 25 (47%) | 1 (2%) | 24 (45%) | 3 (6%) |
| Sally* | 5 | 1 | 4 | 0 | 1 (20%) | 0 | 4 (80%) |
| Tumbo* | 29 | 13 | 16 | 9 (31%) | 4 (14%) | 6 (21%) | 10 (34%) |
| Umugenzi | 27 | 10 | 17 | 8 (30%) | 2 (7%) | 7 (26%) | 10 (37%) |
| Umutama* | 3 | 2 | 1 | 1 (33%) | 1 (33%) | 0 | 1 (33%) |
| Yoyo | 29 | 18 | 11 | 8 (28%) | 10 (34%) | 8 (28%) | 3 (10%) |

| 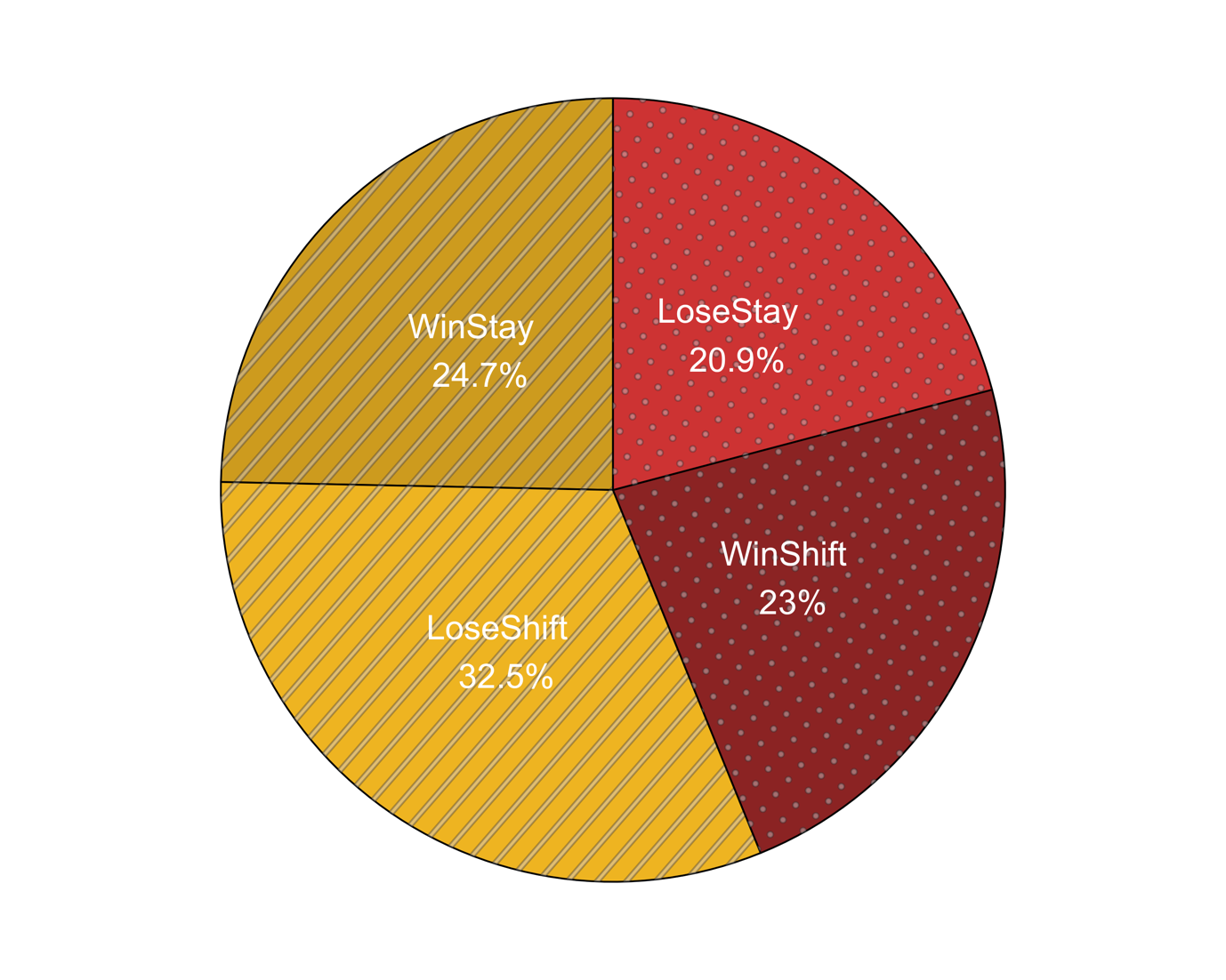 |
| --- |
| **Figure S1** Proportion of using “Win-stay/Lose-shift” and “Win-shift/Lose-stay” strategies after risky choice in the previous trial. |

| Table S3  *Overview of choices after penultimate trial was a risky choice (asterisks indicate individuals who had no side-bias in Experiment 1)* | | | | | | | |
| --- | --- | --- | --- | --- | --- | --- | --- |
| Subject | Total previous risky choices | Previous won | Previous lost | Win-stay | Win-shift | Lose-stay | Lose-shift |
| Asega* | 16 | 11 | 5 | 4 (25%) | 7 (44%) | 1 (6%) | 4 (25%) |
| Baluku* | 0 | 0 | 0 | 0 | 0 | 0 | 0 |
| Becky | 24 | 12 | 12 | 4 (17%) | 8 (33%) | 6 (25%) | 6 (33%) |
| Bili | 30 | 15 | 15 | 8 (27%) | 7 (23%) | 7 (23%) | 8 (27%) |
| Bwambale* | 42 | 21 | 21 | 14 (33%) | 7 (17%) | 18 (43%) | 3 (7%) |
| Cocoa | 30 | 15 | 15 | 8 (27%) | 7 (23%) | 9 (30%) | 6 (20%) |
| Indi | 29 | 15 | 14 | 7 (24%) | 8 (28%) | 8 (28%) | 6 (21%) |
| Kalema | 25 | 11 | 14 | 8 (32%) | 3 (12%) | 6 (24%) | 8 (32%) |
| Kidogo* | 28 | 12 | 16 | 9 (32%) | 3 (11%) | 7 (25%) | 9 (32%) |
| Kisembo | 27 | 12 | 15 | 8 (30%) | 4 (15%) | 5 (19%) | 10 (37%) |
| Mawa* | 5 | 1 | 4 | 0 | 1 (20%) | 0 | 4 (80%) |
| Medina | 38 | 17 | 21 | 14 (37%) | 3 (8%) | 14 (37%) | 7 (18%) |
| Nakuu* | 27 | 11 | 16 | 3 (11%) | 8 (30%) | 9 (33%) | 7 (26%) |
| Namukisa* | 30 | 13 | 17 | 7 (23%) | 6 (20%) | 8 (27%) | 9 (30) |
| Nani* | 9 | 4 | 5 | 1 (11%) | 3 (33%) | 0 | 5 (56%) |
| Nkuumwa* | 5 | 2 | 3 | 0 | 2 (40%) | 1 (20%) | 2 (40%) |
| Pasa* | 17 | 7 | 10 | 2 (12%) | 5 (29%) | 2 (12%) | 8 (47%) |
| Rambo* | 53 | 26 | 27 | 24 (45%) | 2 (4%) | 25 (47%) | 2 (4%) |
| Sally* | 6 | 1 | 5 | 0 | 1 (17%) | 1 (17%) | 4 (67%) |
| Tumbo* | 30 | 14 | 16 | 10 (33%) | 4 (13%) | 7 (23%) | 9 (30%) |
| Umugenzi | 30 | 12 | 18 | 6 (20%) | 6 (20%) | 7 (23%) | 11 (37%) |
| Umutama* | 3 | 2 | 1 | 0 | 2 (67%) | 0 | 1 (33%) |
| Yoyo | 26 | 15 | 11 | 7 (27%) | 8 (31%) | 5 (19%) | 6 (23%) |

**Experiment 2: Individual stability of risky choice**

| Table S4  *Individual stability in Exp. 2. Depicted are average size of the safe option per individual and the proportion of trials within 1.5-point range around these individual means. Individuals are ordered by average size of the safe option.* | | |
| --- | --- | --- |
| Subject | Average size of the safe option  (smaller numbers indicate less risk seeking) | Proportion of trials within 1.5-point range |
| Nani* | 0.9 | 0.95 |
| Umugenzi | 1.2 | 0.95 |
| Mawa* | 1.4 | 0.9 |
| Sally* | 1.4 | 0.9 |
| Baluku* | 1.5 | 0.8 |
| Umutama* | 2 | 0.8 |
| Asega* | 2.1 | 0.95 |
| Kalema | 2.3 | 0.85 |
| Bwambale* | 2.5 | 0.7 |
| Nakuu* | 2.6 | 0.85 |
| Pasa* | 2.8 | 0.95 |
| Nkuumwa* | 3.1 | 0.95 |
| Kidogo* | 3.6 | 0.8 |
| Yoyo | 3.6 | 0.8 |
| Tumbo* | 3.9 | 0.8 |
| Rambo* | 4.2 | 0.9 |
| Medina | 4.35 | 0.8 |
| Kisembo | 4.45 | 0.6 |
| Becky | 4.6 | 0.9 |
| Namukisa* | 4.7 | 0.8 |
| Indi | 4.75 | 0.9 |
| Bili | 5 | 0.85 |
| Cocoa | 5 | 0.9 |

**Effects of sex, age, and trial on choice**

We ran linear models to assess the effect of sex and age on individuals’ risk preference. We checked that the assumption of normally distributed and homogenous residuals was met by inspecting qq plots and the residuals plotted against fitted values. There were no obvious deviations from assumptions. Neither sex nor age predicted risk preferences in Experiment 1 (full sample: *F* = 0.90 , *df* = 2, *p* = .423, *R^2^* = 0.076; subset: *F* = 0.24, *df* = 2, *p* = .794, *R*^2^ = 0.035) and Experiment 2 (full sample: *F* = 1.57, *df* = 2, *p* = .232, *R*^2^ = 0.130; subset: *F* = 0.67, *df* = 2, *p* = .532, *R*^2^ = 0.093).

We ran generalized linear mixed models (GLMM) with binomial error structure to assess the effect of trial on choice. We included z-transformed trial number as a fixed effect and the random slope of trial within subject. We checked that the assumption of normally distributed and homogenous residuals was met by inspecting qq plots and the residuals plotted against fitted values. We checked model stability by comparing the estimates from the model based on all data with those from models with the levels of the random effects excluded one at a time. There were no obvious deviations from assumptions and no indications of model instability. We compared all full models with their respective null model with full random slope structure (using likelihood ratio tests with the *anova* function) to determine if the data was better explained by the latter. Trial number did not predict risk preferences in Experiment 1 (full sample: *χ^2^* = 0.11, *df* = 1, *p* = .746, conditional *R*^2^ = 0.388, subset: *χ^2^* = 0.43, *df* = 1, *p* = .512, conditional *R*^2^ = 0.506).
